# Comparative NanoUPLC-MS^E^ analysis between magainin I-susceptible and -resistant *Escherichia coli* strains

**DOI:** 10.1038/s41598-017-04181-y

**Published:** 2017-06-23

**Authors:** Marlon H. Cardoso, Keyla C. de Almeida, Elizabete de S. Cândido, André M. Murad, Simoni C. Dias, Octávio L. Franco

**Affiliations:** 1 Centro de Análises Proteômicas e Bioquímicas, Pós-Graduação em Ciências Genômicas e Biotecnologia, Universidade Católica de Brasília, Brasília-DF, 70.790-160 Brazil; 2 Programa de Pós-Graduação em Patologia Molecular, Faculdade de Medicina, Universidade de Brasília, Brasília-DF, 70.910-900 Brazil; 3 Embrapa Recursos Genéticos e Biotecnologia, Laboratório de Biologia Sintética, Parque Estação Biológica, Brasília-DF, 70.770-917 Brazil; 4 S-Inova Biotech, Pós-graduação em Biotecnologia, Universidade Católica Dom Bosco, Campo Grande-MS, 79.117-900 Brazil

## Abstract

In recent years the antimicrobial peptides (AMPs) have been prospected and designed as new alternatives to conventional antibiotics. Indeed, AMPs have presented great potential toward pathogenic bacterial strains by means of complex mechanisms of action. However, reports have increasingly emerged regarding the mechanisms by which bacteria resist AMP administration. In this context, we performed a comparative proteomic study by using the total bacterial lysate of magainin I-susceptible and –resistant *E. coli* strains. After nanoUPLC-MS^E^ analyses we identified 742 proteins distributed among the experimental groups, and 25 proteins were differentially expressed in the resistant strains. Among them 10 proteins involved in bacterial resistance, homeostasis, nutrition and protein transport were upregulated, while 15 proteins related to bacterial surface modifications, genetic information and β-lactams binding-protein were downregulated. Moreover, 60 exclusive proteins were identified in the resistant strains, among which biofilm and cell wall formation and multidrug efflux pump proteins could be observed. Thus, differentially from previous studies that could only associate single proteins to AMP bacterial resistance, data here reported show that several metabolic pathways may be related to *E. coli* resistance to AMPs, revealing the crucial role of multiple “omics” studies in order to elucidate the global molecular mechanisms involved in this resistance.

## Introduction

The abusive usage of antibiotics has been considered one of the most important factors that has led to the emergence, positive selection and dissemination of bacterial pathogens resistant to a wide variety of conventional antibiotics applied in animal and human therapies^1^. Both Gram-positive and -negative bacteria have been commonly associated with nosocomial infections in healthcare units. However, even considering the pharmacological and social importance of both groups, the Gram-negative bacteria from clinical isolates have demonstrated an impressive increase antibiotic resistance, mainly the Enterobacteriaceae members such as *Escherichia coli* and *Klebsiella pneumoniae*, which have been related to 95% of infections in healthcare units^2, 3^.

Bacteria have demonstrated diverse biological mechanisms of resistance, which are divided into intrinsic (natural) or acquired^1, 2^. Among them we can cite the direct action of β-lactamase, as well as modified bacterial enzymes in the inactivation of cephalosporin, penicillin, aminoglycosides, gentamicin and streptomycin^2^. In Gram-negative bacteria, studies have shown mechanisms that target the way the drugs are transported, including the selective activity of porins, drug penetration blockage and efflux pumps^4–6^. Moreover, in resistant bacteria the existence has been reported of multi-resistant regions composed of mobile elements such as integrons and transposons, which, once combined, can contribute actively to bacterial resistance^7, 8^.

One of the proposed strategies to avoid the bacterial resistance phenomenon includes the usage of antimicrobial peptides (AMPs). These well-known multifunctional molecules have been widely prospected from several organisms^9–11^, also acting as mother molecules for rational design approaches^12^. Moreover, AMPs have gained more and more attention due to their efficacy in inhibiting multidrug-resistant strains by complex mechanisms of action involving membrane destabilization and disruption, interference in several biosynthetic pathways, generation of reactive oxygen species and enzyme inhibition^9, 12, 13^. Among AMPs is the cathelicidin family, including 30 members such as cecropin^14^, bactenecins^15^ and magainins^16^. Magainins, the object of our study, are cationic, amphipathic helical peptides, with 23 amino acid residues in length, which were first isolated from the African frog *Xenopus laevis* ^16^. These peptides are divided into two subfamilies encoded by the same gene, denominated magainin I and magainin II, which differ in their primary sequence at positions 10 and 22 (Gly^10^ and Lys^17^ for magainins I; Lys^10^ and Asp^17^ for magainins II)^17, 18^. Studies have shown that magainins mainly interact with negatively charged acidic phospholipids, which are more characteristic of prokaryotic membranes^19^. Furthermore, studies have related that magainin’s amphiphilic α-helical structure can form ion-permeable channels, resulting in membrane depolarization, irreversible cytolysis and finally leading to cell death^20^. Allied to such properties, magainin peptide analogues (e.g. pexiganan®, an antibacterial drug) represent a select group of AMPs, with more than 1,158 patents deposited in the US Patent Ful-Text Database Boolean Search server. This group of AMPs were evaluated in advanced clinical trials and submitted for the approval of the Food and Drug Administration (FDA), thus revealing the importance behind bacterial resistance to these cathelicidin members.

Although the above-mentioned AMPs seem to be promising alternatives to conventional antibiotics we cannot discard the fact that studies have already shown that bacteria are able to respond to AMPs by different strategies. Among them is the expression of extracellular proteins such as metallo-, serine- and cysteine proteinases involved in AMP degradation^21, 22^. In addition, the expression of exopolymers such as polysaccharide intracellular adhesin (PIA) and capsular polysaccharides (CPS) has been described as a strategy to capture or repel AMPs^23, 24^. It was observed that nontypeable *Haemophilus influenzae* Gram-negative strains can express Sap (sensitivity antimicrobial peptides) ABC transporters to resist *in vitro* and *in vivo* AMP treatment^25^. Interestingly, it has been suggested that a series of Sap transporters may act in a potassium-dependent manner in order to deliver AMPs to the bacterial cytoplasm for further intracellular degradation^25, 26^. Since the first contact with the bacterial membranes is one of the initial steps for AMP action, some strains have also evolved the capacity of modifying the physicochemical properties of their surfaces and cytoplasmic membrane, preventing AMP electrostatic binding^27, 28^. Finally, even when AMPs have overcome the initial physical barriers (cell wall and membranes), reaching the intracellular environment, resistant bacteria are able to expel them by efficient efflux pumps^29, 30^. However, so far, although such mechanisms of resistance have been elucidated, only a few works have explored the molecular networks involving omics technologies in order to explain the development of resistance to AMPs.

We have previously reported the evaluation of the cytosolic subproteome of magainin I-resistant *E. coli* strains by 2D electrophoresis allied to tandem mass spectrometry *de novo* sequencing. However, in addition to this approach, the usage of high-throughput strategies such as nanoUPLC-MS^E^ has been reported in the literature as an important technique used to trace the global functional profile of bacteria, allowing us to analyze how these microorganisms are able to trace routes in order to escape from antimicrobial agents^31, 32^. Thus, here we focused on a deep comparative nanoUPLC-MS^E^ analysis by using the total lysate extract from *E. coli* strains that are susceptible and resistant to the AMP magainin I, evaluating the differential expressed, as well as exclusive proteins in order to shed some light on the global molecular mechanisms involved in this resistance.

## Results

### Protein identification and classification

The proteins from the total bacterial lysate of *E. coli* (ATCC 8739), control magainin I-susceptible (C1-3) and -resistant (R1-3) strains were obtained according to the experimental procedure previously described in the methodology section. After strain selection, a total proteinaceous yield of 24–29 mg was obtained. Further, nanoUPLC-MS^E^ was applied in order to compare the proteomic profile of the total bacterial lysate for all strains, identifying the significant differences between the experimental groups ATCC, magainin I-susceptible and -resistant strains cultured under optimal growth conditions.

**Figure 1:**
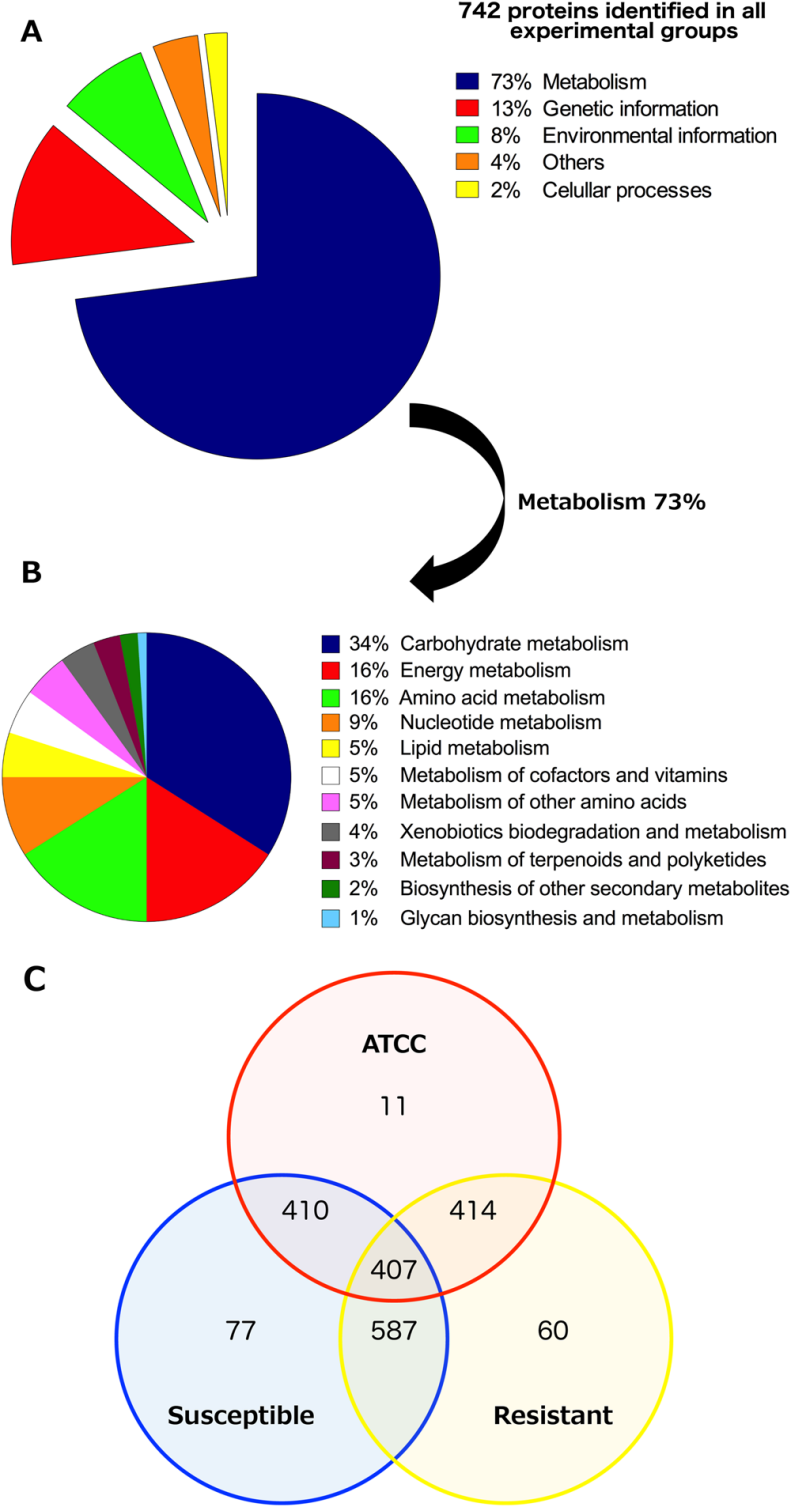
*E. coli* protein classification according to KEGG biologic classes. (**A**) Biological classes assigned for a total of 742 proteins identified in all experimental groups; (**B**) detailed distribution of proteins related to the bacterial metabolism; (**C**) Venn diagram showing the proteins identified through nanoUPLC-MS^E^, correlating the ATCC 8739, magainin I–susceptible and –resistant groups.

After data processing, it was possible to identify 742 proteins distributed among the experimental groups, 427 proteins being identified in the *E. coli* (ATCC 8739) group, 664 proteins in the *E. coli* susceptible (control) group and 651 proteins identified in the *E. coli* resistant group. In order to build a protein functional profile, the identified proteins were separated into categories and subcategories using KEGG Orthology (KOs). The obtained KO assignments were distributed into five biological classes (Fig. 1A). According to the database, the biological classes assigned were cellular processes (2%), environmental information processing (8%), genetic information processing (13%), metabolism (73%) and others (4%) (Fig. 1A). As metabolism was the most representative class (representing 73% of the total proteins identified), subcategories were also evaluated and were classified as carbohydrate (34%), energy (16%), amino acid (16%) and nucleotide metabolisms (9%) (Fig. 1B).

### Common, differential and unique proteins between all groups

**Figure 2:**
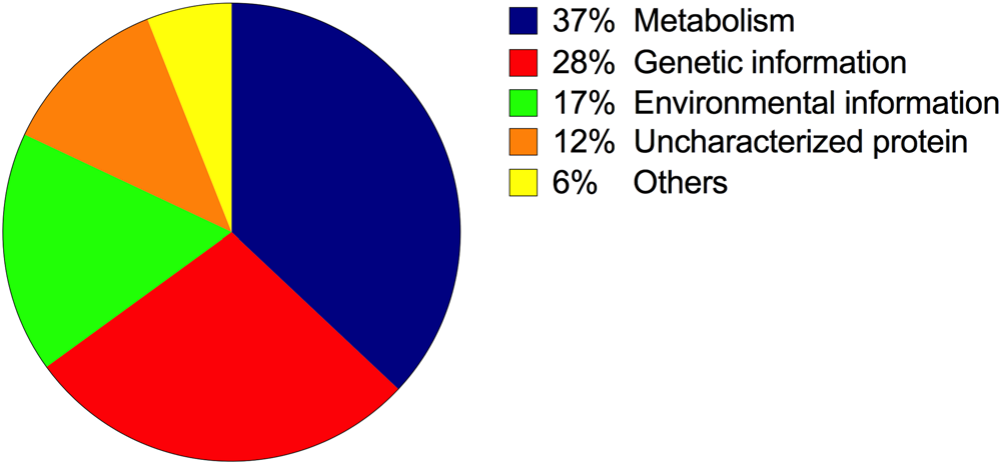
*E. coli* magainin I–resistant exclusive protein classification. Graphical distribution of *E. coli* magainin I–resistant exclusive proteins according to KEGG biological classes.

Proteins of each experimental group were compared in order to identify the common and exclusive proteins among them. It was possible to identify 77, 11 and 60 exclusive proteins in the susceptible, ATCC and resistant groups, respectively (Fig. 1C). Among the 60 exclusive proteins from the magainin I-resistant group, 37% could be related to metabolism, 28% to genetic information and 17% to environmental information, while 12% were uncharacterized proteins and 4% others (Table S1 and Fig. 2). Regarding the shared proteins, the Venn diagram showed that 407 identified proteins were common to the three groups analyzed (Fig. 1C). Observing the intersections in pairs it was possible to verify that 410 proteins were shared between the susceptible and ATCC group, 414 proteins between the resistant and ATCC groups and, last, 587 proteins were common between the susceptible and resistant groups (Fig. 1C). Furthermore, among the 587 proteins identified and shared by the magainin I-susceptible and magainin I-resistant groups, according to the parameters described before, 25 proteins were identified with differential expression in the magainin I-resistant group in comparison with the control group (Table 1).

**Table 1:** Comparison of protein expression between magainin I-susceptible and magainin I-resistant *Escherichia coli* strains identified via nanoUPLC-MS^E^ and their participation in the bacterial biological process.

Accession Number	UniProt Identification	Protein Expression	Score	Log (e) Ratio^*^	Variance^*^	Protein Description	Molecular Mass (Da)	Biological Classes
P0A915	OMPW_ECOLI	Upregulated	236.44	0.50	0.45	Outer membrane protein W	2292.8	Environmental Information Processing
P06996	OMPC_ECOLI	Upregulated	3576.12	0.65	0.03	Outer membrane protein C	4036.8	Environmental Information Processing
P02931	OMPF_ECOLI	Upregulated	639.19	0.78	0.07	Outer membrane protein F	3933.3	Environmental Information Processing
Q1PI90	Q1PI90_ECOLI	Upregulated	2207.53	0.71	0.04	Outer membrane protein 1b (Fragment)	2759.4	Environmental Information Processing
P75678	YKFA_ECOLI	Upregulated	283.62	0.52	0.51	Uncharacterized protein	3189.2	Uncharacterized protein
P23843	OPPA_ECOLI	Upregulated	558.97	0.67	0.05	Periplasmic oligopeptide-binding protein	6089.9	Environmental Information Processing
P39165	YCHO_ECOLI	Upregulated	387.49	0.68	0.33	Uncharacterized protein	5210.0	Uncharacterized protein
P0AAA9	ZRAP_ECOLI	Upregulated	14392.98	0.70	0.05	Zinc resistance-associated protein	1519.9	Environmental Information Processing
P03841	MALM_ECOLI	Upregulated	311.79	0.74	0.22	Maltose operon periplasmic protein	3194.3	Environmental Information Processing
P0ADE6	YGAU_ECOLI	Upregulated	772.28	0.80	0.13	Uncharacterized protein	1606.3	Uncharacterized protein
P13033	GLPB_ECOLI	Downregulated	483.13	2.03	0.36	Anaerobic glycerol-3-phosphate dehydrogenase subunit B	4535.7	Metabolism
P09394	GLPQ_ECOLI	Downregulated	2564.38	1.40	0.18	Glycerophosphoryl diester phosphodiesterase	4084.3	Metabolism
P0A9C0	GLPA_ECOLI	Downregulated	2024.03	1.28	0.18	Anaerobic glycerol-3-phosphate dehydrogenase subunit A	5895.8	Metabolism
P0AB14	YCCJ_ECOLI	Downregulated	42340.24	0.80	0.07	Uncharacterized protein	8524.0	Uncharacterized protein
P76268	KDGR_ECOLI	Downregulated	403.70	0.79	0.30	Transcriptional regulator kdgR	3002.9	Genetic Information Processing
P64463	YDFZ_ECOLI	Downregulated	12233.5	0.77	0.05	Putative selenoprotein ydfZ	7276.0	Metabolism
P77454	GLSA1_ECOLI	Downregulated	485.06	0.74	0.17	Glutaminase 1	3290.3	Metabolism
P0AAS7	YBCJ_ECOLI	Downregulated	854.17	0.60	0.37	Uncharacterized protein	7390.0	Uncharacterized protein
P0AGL2	TDCF_ECOLI	Downregulated	826.76	0.57	0.16	Protein tdcF	1400.7	Metabolism
Q59385	COPA_ECOLI	Downregulated	205.89	0.53	0.36	Copper-exporting P-type ATPase A	8787.3	Environmental Information Processing
P0A6K3	DEF_ECOLI	Downregulated	392.11	0.52	0.46	Peptide deformylase	1932.8	Metabolism
P0A759	NAGB_ECOLI	Downregulated	276.4	0.51	0.37	Glucosamine-6-phosphate deaminase	2977.4	Metabolism
P0ABT2	DPS_ECOLI	Downregulated	32750.34	0.51	0.03	DNA protection during starvation protein	1869.5	Genetic Information Processing
P0A6Y8	DNAK_ECOLI	Downregulated	19211.09	0.51	0.02	Chaperone protein DnaK	6911.5	Genetic Information Processing and Metabolism
P0A8G6	WRBA_ECOLI	Downregulated	11264.48	0.50	0.03	Flavoprotein wrbA	2084.6	Genetic Information Processing and Metabolism

*Protein differential expression was considered significant in a Log(e) ratio ≥0.5 and a variance value ≤0.51.

### Differential proteins in the magainin I-resistant group

Shared proteins between the magainin I-resistant and -susceptible control were analyzed with regard to their differential expression. It was observed that among the 25 identified proteins with differential expression, 10 were upregulated, mostly related to environmental biological processes; while 15 were downregulated, mainly participating in carbohydrate, amino acid, other amino acid and energy metabolism, as well as in genetic information processing such as DNA replication, transcription and repair (Table 1; Figs S2 and 3). Among 10 upregulated proteins, 3 were uncharacterized proteins (YKFA_ECOLI, YCHO_ECOLI and YGAU_ECOLI) and 4 proteins were outer membrane proteins such as W (OMPW_ECOLI), C (OMPC_ECOLI), F (OMPF_ECOLI) and 1b fragment (Q1PI90_ECOLI). Moreover, the periplasmic oligopeptide-binding protein (OPPA_ECOLI), the zinc resistance-associated protein (ZRAP_ECOLI) and the maltose operon periplasmic protein (MALM_ECOLI) were also identified (Table 1; Figs S2 and 3).

**Figure 3:**
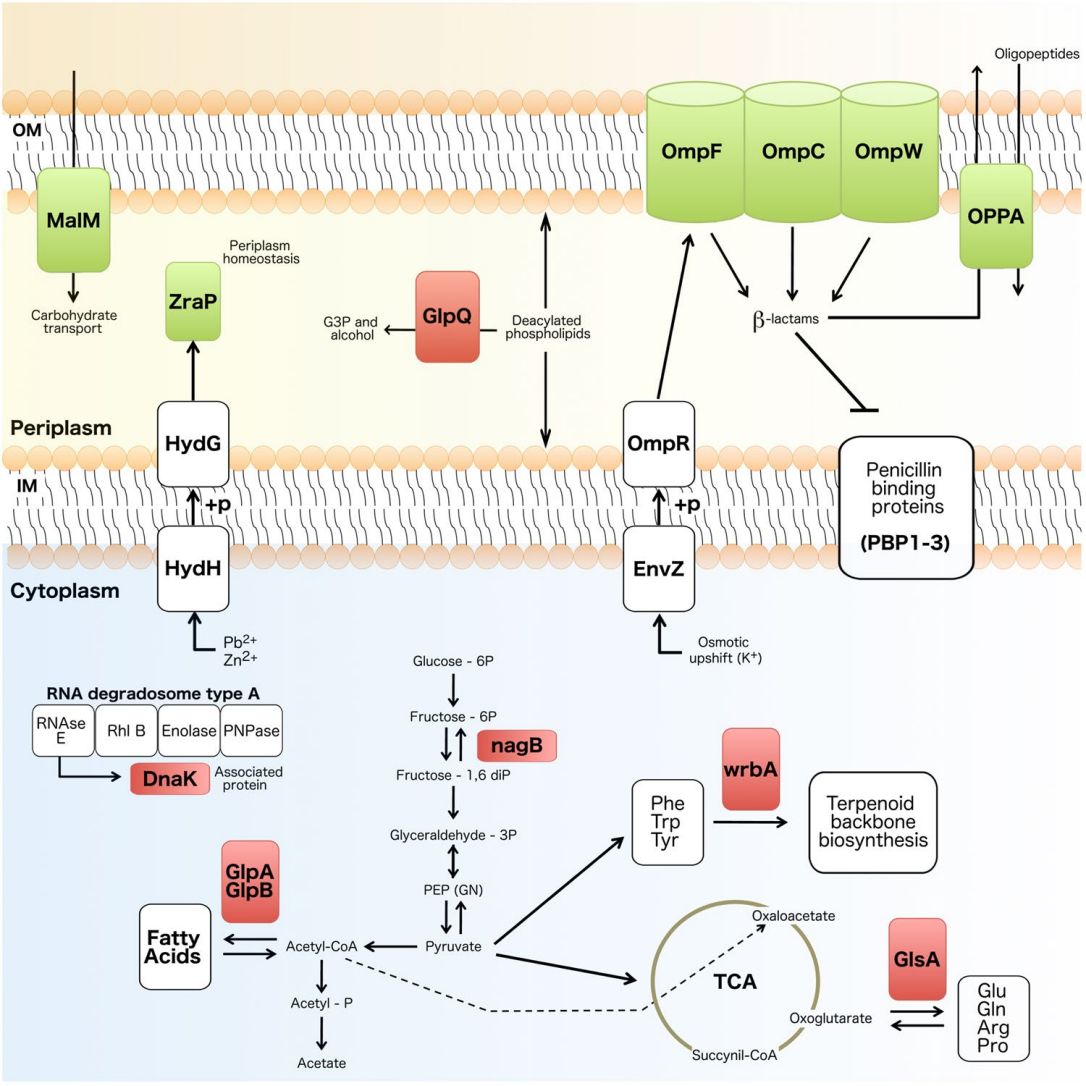
Schematic magainin I-resistant *E. coli* cell representation. Schematic representation of a magainin I-resistant cell, highlighting its differentially expressed proteins (green: upregulated; red: downregulated), as well as their biological pathways according to KEGG. Differentially expressed proteins observed in Table 1 and not represented here did not present biological pathways on KEGG. Abbreviations: OM: outer membrane; IM: inner membrane; OmpC, F and W: outer membrane proteins; MalM: maltose operon periplasmic protein; ZraP: zinc resistance-associated protein; GlpQ: glycerophosphoryl diester phosphodiesterase; GlpA and B: anaerobic glycerol-3-phosphate dehydrogenase subunit A and B, respectively; G3P: glycerol-3-phosphate; OppA: periplasmic oligopeptide-binding protein; HydH: sensor protein ZraS; HydG: transcriptional regulatory protein ZraR; OmpR: transcriptional regulatory protein OmpR; EnvZ: osmolarity sensor protein EnvZ; DnaK: chaperone protein DnaK; NagB: glucosamine-6-phosphate deaminase; WrbA: NAD(P)H dehydrogenase (quinone); glutaminase 1; Phe: phenylalanine; Tpr: tryptophan; Tyr: tyrosine; Glu: glutamic acid; Gln: glutamine; Arg: arginine; Pro: proline; TCA: tricarboxylic acid cycle.

Furthermore, among the 15 downregulated proteins identified in the resistant strain, two were uncharacterized proteins (YCCJ_ECOLI and YBCJ_ECOLI). The other 13 downregulated proteins were annotated as anaerobic glycerol-3-phosphate dehydrogenase subunits A (GLPA_ECOLI) and B (GLPB_ECOLI), glycerophosphoryl diester phosphodiesterase (GLPQ_ECOLI), putative selenoprotein ydfZ (YDFZ_ECOLI), glutaminase 1 (GLSA1_ECOLI), protein tdcF (TDCF_ECOLI), peptide deformylase (DEF_ECOLI), glucosamine-6-phosphate deaminase (NAGB_ECOLI), transcriptional regulator kdgR (KDGR_ECOLI), DNA protection during starvation protein (DPS_ECOLI), chaperone protein DnaK (DNAK_ECOLI), flavoprotein WrbA (WRBA_ECOLI) and copper-exporting P-type ATPase A (COPA_ECOLI) (Table 1; Figs S2 and 3). All codes represent UniProt entries.

## Discussion

Because the threat of bacterial resistance is of great concern nowadays, it is clearly extremely urgent to acquire more detailed molecular information regarding the mechanisms by which resistant bacteria escape from AMPs. With that in mind, here we reported a comparative proteomic study between *E. coli* strains that are susceptible and resistant to the well-studied AMP magainin-I. After induction of resistance, as well as molecular identification of resistant strains, the total bacterial lysates were submitted to nanoUPLC-MS^E^ analyses. A total of 742 proteins were identified, 73% being related to bacterial metabolism. Also, 407 proteins were common to all groups studied. Moreover, from the 587 proteins shared by magainin I-susceptible and –resistant strains, 25 proteins could be identified with differential expression patterns, and 10 were upregulated (3 uncharacterized) and 15 downregulated (2 uncharacterized). Furthermore, 60 proteins were found to be exclusive to magainin I-resistant strains.

Among the upregulated proteins, three were characterized as outer membrane proteins (OMPs) and three as periplasmic transport proteins involved in bacterial resistance, homeostasis and nutrition. The OMPs were here represented by OmpW, OmpC and OmpF. This class of proteins has been well described as porins responsible for creating a size-selective channel in order to transport small hydrophilic molecules, including some classes of antibiotics^33^. In *E. coli* the presence of OmpC and OmpF has been related to the resistance of this strain to β-lactams^34^. Also, some studies have shown that OmpW, when associated with the small multidrug resistance protein member (EmrE), facilitates the efflux of toxic quaternary cationic compounds^35^. Interestingly, in contrast to what we observed in our work, some studies have reported that a downregulation in the major pore proteins, including OmpC, OmpF and OmpW, favors bacterial resistance^36–38^. However, works regarding *E. coli* porin expression have revealed that OmpW and OmpC were upregulated in tetracycline and nalidixic acid resistant strains^39, 40^. All these findings might indicate an antibiotic-specific pattern of porin expression, which can also be associated with bacterial resistance to different classes of AMPs.

An oligopeptide-binding protein (OppA) was also upregulated. OppA is located at the periplasmic space and participates in different biological processes including oligopeptide and protein transport, as well as response to heat. Studies performed with clinical *E. coli* (K12) have shown that decreasing the levels of OppA or inducing its depletion leads to this strain becoming resistant to aminoglycoside antibiotics^41^. Interestingly, more recently, it was reported that two different members from the aminoglycoside class, amikacin and neomycin, might contribute differentially in the expression level of OppA in *E. coli-*resistant strains, where the first induces the downregulation and the second the upregulation of this protein^42^. Due to these divergent data, two hypotheses have appeared, suggesting that a downregulation of OppA might hinder the entrance of antibiotics, leading to resistance, or that its upregulation probably facilitates the transport of antimicrobial agents to the cytosol for proteolytic degradation. The latter hypothesis has been more accepted in the field of AMPs, including in our work, where different ABC transporters are described to participate in the proteolytic pathway of these molecules^25, 43^.

A zinc resistance-associated periplasmic protein (ZraP) was also found to be upregulated in our magainin I-resistant strains. ZraP is produced in the presence of Zn^2+^ and Pb^2+^, and it is also associated with envelope homeostasis, as well as chaperon and regulatory functions^44, 45^. Studies have also highlighted the importance of ZraP in bacterial strains submitted to envelope stress, sometimes caused by the presence of bactericidal agents such as AMPs. When pore-forming AMPs (eg. magainin) display their activities on bacterial surfaces, an increase in the production of ZraP is expected in order to maintain the periplasmic space homeostasis, providing an optimal environment for other resistance-related enzymes and proteins^44^. In addition, such stress response due to antibiotic/AMP presence has also been shown to induce magainin I-resistant strains to invest in proteins involved in energy metabolism, such as the maltose operon protein (MalM), which was also upregulated in this study. MalM is poorly studied and its function is as yet unclear. However, it is known that MalM is expressed as a part of the *malK-lamB* operon, perhaps acting as a carbohydrate transporter and, thus, assisting bacterial nutrition during stress conditions, in this case, magainin I presence^46^.

Among the downregulated proteins, seven could be distributed into biological pathways, where three participate in the phospholipid metabolism and four in the processing of genetic information. Those related with phospholipids were divided into two classes of enzymes, a periplasmic glycerophosphoryl diester phosphodiesterase (GlpQ), and two cytosolic anaerobic glycerol-3-phosphate dehydrogenases (GlpA and GlpB). One of the strategies that bacteria adopt to escape from AMPs is modifying the physicochemical surface properties, affecting AMP attachment^28^. This modification may be intermediated by the acetylation of phospholipids^47^. In this situation GlpQ appears, which is capable of hydrolyzing deacylated phospholipids into glycerol-3-phosphate and alcohol^48^. Allied to that, in the intracellular environment GlpA and GlpB, jointly with GlpC, form the enzymatic complex (GlpA/B/C/) responsible for converting dihydroxyacetone phosphate into glycerol-3-phosphate, a precursor in the synthesis of phospholipids^49^. Thus, reports have shown that differential expression of GlpQ, GlpA and GlpB assists the modification and distribution of phospholipids within bacterial membranes, also favoring bacterial resistance to AMPs and antibiotics^49, 50^. Furthermore, in the case of pore-forming AMPs, including magainin I, such membrane modifications are of great importance, since they can divert these molecules away from their main target.

In addition to cytosolic GlpA and GlpB, we also identified the biological pathways for a chaperone protein DnaK, a glutaminase 1 (glsA1), a glucosamine-6-phosphate deaminase (NagB) and a NAD(P)H dehydrogenase (WrbA). As reported in our previous work^32^, here we also observed alterations in the patterns of expression of DnaK in a non-stress environment. DnaK is involved in several biological processes, including DNA replication, response to heat, and protein folding^51–53^. Moreover, the levels of DnaK have been evaluated in different works regarding bacterial exposure to antimicrobial agents, also indicating the crucial role of this protein for bacterial survival under stress conditions due to its ability to capture protein aggregates accumulated in bacteria during antibiotic/AMP treatment^51^. Also, in response to stress, bacteria can produce NAD(P)H dehydrogenase (WrbA), which is supposed to reduce quinones into their hydroquinone states^54, 55^. This reaction prevents the interactions between semiquinones and O_2_, also avoiding the formation of reactive oxygen species such as superoxide, which are toxic to bacterial cells^54^. In addition, the alteration in the expression of a glutaminase 1 (GlsA1) also indicates bacterial adaptation after AMP exposure. Indeed, GlsA1 is known to catalyze the hydrolytic deamination of L-glutamine in L-glutamate and NH^4+^ ^56^. Besides, it has been reported that glutaminases (eg. GlsA) belong to the group of serine β-lactams and penicillin-binding proteins and, for this reason, represent an important class of molecules in the advent of bacterial resistance^57, 58^.

Carbon is an essential compound for the biosynthesis of several bacterial molecules, including those in response to antibiotics/AMPs. Here, the differential expression of a glucosamine-6-phosphate deaminase (NagB) was also observed. Studies have shown that NagB is able to catalyze the deamination and isomerization of D-glucosamine-6-phosphate reversely into D-fructose 6-phosphate and an ammonium ion^59^. Interestingly, in *E. coli* strains it was found that NagB is activated by N-acetyl-D-glucosamine-6-phophate, further participating in the catabolism of amino sugar, facilitating the use of glucosamine and N-acetyl-D-glucosamine as carbon sources by bacteria^59^.

Finally, downregulated proteins that could not be allocated in any biological pathways in our analyses may be involved in amino acid degradation, copper export, DNA protection and transcriptional regulator. Moreover, 60 exclusive proteins were identified in the magainin I-resistant strains, mostly related with metabolism and processing of genetic information. Among them, it is important to highlight the presence of proteins related to biofilm formation (YoaB) and cell wall formation (DblB). Biofilms are of great concern nowadays, representing 65 to 85% of all human infections^60^. Also, it has been proved that bacteria in their biofilm state may present 100 to 1000-fold increased resistance to antibiotics^61^. The presence of a biofilm-related protein (YoaB) in our magainin I-resistant strains clearly demonstrates an adaptive strategy to escape from magainin I by improving physical and chemical barriers. The peptidoglycan layer also consists of a physical barrier that bacteria developed for their defense. The protein D-alanine:D-alanine ligase (DblB) is an enzyme involved in the production of one of the components of peptidoglycan^62^. DblB was here identified as exclusive to magainin I-resistant strains. In the case of AMPs overcoming bacterial physical barriers, bacteria can also express efflux pump proteins in order to expel AMPs from the periplasmic and cytosolic spaces. Here a multidrug efflux pump subunit (AcrA)^63^ was also exclusive to magainin I-resistant strains, once more indicating how complex and diverse the mechanisms of resistance are that bacteria adopt in response to AMPs.

Differentially from previous studies that only associated single proteins with bacterial resistance to AMPs^21, 30^, the exploratory data here reported, using protein identification and quantitative assessment (Log(e) ratio and variance), suggest that several metabolic pathways may be related to *E. coli* resistance to AMPs. These data provide global molecular information that could further support biochemical studies of the resistance-related proteins identified. In general, the bacterial response observed here illustrates the process of adaptive resistance present in an environment that is non-hostile to bacterial growth conditions, such as ideal conditions of temperature, pH, source of nutrition, aeration and without the negative pressure of AMPs. Furthermore, we have shown that *E. coli* resistant to magainin I developed a complex and diversified molecular mechanism of adaptations and bacterial defense, also indicating that the mechanism of action of this peptide appears to be much more complex than just the interference in the stability of the bacterial cell membrane. Moreover, it is important to highlight the relevance and urgency for more studies that involve analyses using proteomics, transcriptomics, and genomics in order to further elucidate the complete panel of bacterial resistance to AMPs.

## Material and Methods

### Magainin I synthesis

The peptide magainin I was purchased from Peptide 2.0 Incorporated (USA) at 95% purity (after TFA removal) by the stepwise solid-phase method using the N-9-fluorenylmethyloxycarbonyl (Fmoc) chemistry on a Rink amide resin.

### Bacterial strains

*E. coli* (ATCC 8739) was used to prepare the magainin I-susceptible and -resistant strains from 10 successive propagations in Luria-Bertani (LB) medium in the absence or presence of magainin I at 0.5 × minimum inhibitory concentration (MIC) in order to induce magainin resistance, as previously described^32^. For this, a pre-inoculum was prepared from an *E. coli* strain (ATCC 8739) in LB broth at 37 °C, for 16 h, with stirring at 240 rpm. Subsequently, 100 μL of the pre-inoculum was added to 2.5 mL of LB broth at 37 °C and 240 rpm, until the middle of its exponential phase was reached by monitoring the growth at 595 nm. In order to induce resistance to magainin I, 5 × 10^5^ CFU.mL^−1^ of this inoculum was transferred to 100 mL of LB in the presence of magainin I 0.5 × MIC and incubated for 2 h, at 37 °C and 240 rpm. This procedure was repeated in 10 successive propagations under the same conditions. The bacterial suspension resulted from the last propagation was then spread overnight on a magainin I-free LB agar plate at 37 °C. Three biological and technical replicates were experimentally conducted, and a single magainin I-resistant colony was selected for each replicate. In addition, the entire experiment was also performed in the absence of magainin I as a negative control in the resistance procedures in order to minimize further observation of differential expressed proteins due to divergent bacterial manipulation and experimental conditions. Thus, the resulting magainin I-resistant (R1, R2 and R3), and the control magainin I-susceptible colonies were named (C1, C2 and C3) and were defined. Strain identification and antimicrobial susceptibility assays (VITEK and MicroScan) were performed showing the specific resistance of *E. coli* (ATCC 8739) to magainin I, and published elsewhere^64^. In addition to the R1-3 and C1-3 groups, the original *E. coli* (ATCC 8739) was used for further comparative proteomics analyses as a control not submitted to any resistance procedures.

### Preparation of total bacterial lysate

Bacterial inoculums of the original *E. coli* (ATCC 8739), the susceptible (C1, C2 and C3) and the magainin I-resistant strains (R1, R2 and R3) were cultured in 50 mL of LB medium at 37 °C, for 16 h, with stirring at 240 rpm. The optical density (O.D.) of endpoint bacterial growth was measured at 595 nm. The bacterial suspensions of all strains were inoculated to a final concentration of 5 × 10^5^ CFU. mL^−1^ (0.05 AU at 595 nm) in 2 L of LB broth at 37 °C, for 3 h, with stirring at 240 rpm. After time incubation, the suspensions were immediately centrifuged at 4 000 *g*, 4 °C, for 10 min, and the supernatants were discarded. The pellets were re-suspended in phosphate buffered saline and incubated with protease inhibitor cocktail in a 1:100 ratio (v/v).

### Protein sample precipitation

The bacterial suspensions were submitted to cell lysis by sonication in cold for 60 s and 40 A, for 15 cycles. Then the bacterial lysates containing the total protein samples were centrifuged at 4 000 *g*, 4 °C, for 30 min, and the supernatants were recovered. These supernatants were used for protein precipitation with cold 100% acetone in a 3:1 ratio (v/v) at 4 °C, for 16 h. After time incubation, the samples were centrifuged at 4 000 *g*, 4 °C, for 10 min, and the pellets were re-suspended in phosphate buffered saline. The protein samples were quantified at 750 nm using the RCDC protein assay kit (BioRad).

### Protein sample preparation for nanoUPLC-MS^E^ acquisition

The suspensions containing the total protein samples from bacterial lysate of *E. coli* (ATCC 8739), and magainin I-susceptible and -resistant *E. coli* strains were digested with trypsin enzyme (Promega) for acquisition in a nanoUPLC-MS^E^. Briefly, 100 μg of each protein sample was mixed with 50 mM ammonium bicarbonate and 0.2% RapiGest SF solution (Waters). The mixture was homogenized and incubated at 80 °C, for 15 min. Then, 100 mM dithiothreitol was added and the samples were incubated at 60 °C, for 30 min. After that, the samples were briefly centrifuged and 300 mM iodoacetamide was added, remaining incubated at room temperature, for 30 min and protected from light. After time incubation, trypsin enzyme (Promega) was added in a 1:100 ratio (v/v) and the samples were incubated at 37 °C, for 16 h for time digestion. For surfactant hydrolysis and precipitation, 5% TFA was added at 37 °C, for 90 min. The samples were centrifuged at 14,000 *g*, 6 °C, for 30 min and the supernatants were recovered and lyophilized. After that, the samples were re-suspended in 190 µL of 20 mM of ammonium formate (Sigma-Aldrich). Also, 10 µL of MassPREP^TM^ digestion standard Phosphorylase b (Waters) was added (stock 1pmol.μL^−1^) as a standard of protein digestion (final concentration of 50 fmol.μL^−1^). The sample preparation was performed in triplicate.

### NanoUPLC-MS^E^ acquisition

The acquisition in LC-MS was performed in accordance with Murad and co-workers^65^, with modifications. Nanoscale LC separation of tryptic peptides was performed using the nanoACQUITY™ system (Waters) via bidimensional technology by dilution. The first dimension was performed using an XBridge™ nanoEase™ BEH130 C18 5 μm, 300 mm × 50 mm column (Waters). Mobile phase A was 20 mM ammonium formate and mobile phase B was acetonitrile. The second dimension was performed by using a Symmetry C18 5 μm, 5-mm × 300-μm analytical reversed-phase pre-column (Waters) and a HSST3 C18 1.8 μm particle, 75 μm × 150 mm analytical reversed-phase column (Waters). Mobile phase A was 0.1% formic acid in water and mobile phase B was 0.1% formic acid in acetonitrile. Initially, 5 µL of sample was transferred to the first dimensional column using 0.1% acetonitrile with a flow rate of 2 μL.min^−1^, for 0.5 min. The peptides from the first fraction were eluted using 10.8% mobile phase B for 2 min with a flow rate of 2 μL.min^−1^ and then diluted in the pre-column using an aqueous 0.1% formic acid solution with a flow rate of 20 μL.min^−1^, for 8.5 min. The peptides were separated using a gradient of 7–35% mobile phase B for 37 min with a flow rate of 500 ηL.min^−1^ followed by a 5 min rinse with 85% of mobile phase B. The column was re-equilibrated to the initial conditions for 10 min. The column temperature was maintained at 35 °C. The sample injection was programmed to apply the exact volume of the injection loop. The peptides from the second, third, fourth and fifth fractions were eluted using 14%, 16.7%, 20.4% and 50% of mobile phase B, respectively. The dilution, flow rate and separation conditions were maintained for all fractions in the second dimension. The lock mass Glu-fibrinopeptide (GFP) was delivered from the reference pump of the NanoLockSpray using a SynaptG2 system with a constant flow rate of 500 ηL.min^−1^ at a concentration of 320 fmol of GFP. All samples were analyzed in triplicate. The tryptic peptides were analyzed using a Synapt G2 HDMS™ mass spectrometer (Waters, Manchester, UK) in positive mode, using a nanoSpray and with a resolution power of at least 20000 full-width half-maximum (FWHM). The time-of-flight analyzer of the mass spectrometer was externally calibrated with GFP b+ and y+, m/z ranging from 50 to 2000, and data post-acquisition lock mass corrected using the m/z from the GFP double charged precursor ion (GFP [M + 2H]^2+^ = 785.8426). The reference sprayer was sampled at a frequency of 30 s and the exact mass retention time (EMRT) nanoLC-MS^E^ data were collected in an alternating independent low- and high-energy acquisition mode. EMRT was de-isotoped and charge state-reduced and then was replicated throughout the complete experiment for the analysis at EMRT cluster level (Figure S1). The continuum spectra acquisition time was 1.5 s with a 0.1 s interscan delay, in each mode. In the low-energy MS mode the data were collected at 3 eV constant collision energy, and in the elevated energy MS mode the collision energy was ramped from 12 to 45 eV during each 1.5 s scan. The quadrupole mass analyzer radiofrequency was adjusted so that the ions from m/z 400 to 2000 were efficiently transmitted, ensuring that any ions less than m/z 400 that were observed in the LC-MS data only arose from the dissociations in the T-WAVE cell.

### LC-MS data processing and protein identification

The MS data obtained from the LC-MS^E^ were processed and searched using the ProteinLynx Global Server (PLGS) software version 3.0 (Waters). The protein identification was mediated by the software’s embedded ion accounting algorithm^66, 67^ via comparison with the *E. coli* database, including the sequences from the MassPREP digestion standards (MPDS) Phosphorylase-B. For protein quantification, a comparison was performed between the intensity rates measured for three prototype peptides of each protein with the intensity rates of three prototype identified peptides of the digested internal standard using dedicated algorithms^68^. The protein identification criteria included: (1) the detection of at least one fragment ion per peptide; (2) three fragment ions per protein; (3) one peptide per protein; (4) 4% maximum rate of false-positive; (5) 250 fmol of the protein calibration P00489; (6) variable modifications including acetyl N-terminus, N deamination, Q deamination, M oxidation; (7) specification of the carbamidomethyl-C fixed modification; (8) trypsin digestion allowing only one cleavage error. The common, exclusive and differential (upregulated or downregulated) proteins were separated in each group using the PLGS software with an Expression^E^ license installed using a logarithm rate of the protein concentration in fmol and with data normalization. The *p* value was used to indicate if the protein expression is upregulated (*p* > 0.95) or downregulated (*p* < 0.05). Any values different from these extremes indicated no protein expression alteration. The proteins expressed in the biological and the technical replicates in all groups were considered common proteins, and the proteins expressed only in a specific group were considered exclusive proteins. The proteins expressed in the biological and the technical replicates in all groups were considered common proteins, and the proteins expressed only in a specific group were considered exclusive proteins. As regards the differential protein expression, two more parameters were also considered for quantitative assessment. First we used fold change value [Log(e) ratio] to determine how much the protein expression was increased or decreased between two groups. Then a variance value was utilized, indicating how much a protein varied between the biological and the technical replicates of each group. Thus, only those proteins that showed a differential expression with a Log(e) ratio ≥0.5 and a variance ≤0.51 were considered significant. In addition, the proteins of each group were distinguished according to their cellular function using the Kyoto Encyclopedia of Genes and Genomes (KEGG, http://www.genome.jp/kegg/) database to search for possible molecular markers of bacterial resistance.

## Electronic supplementary material

Figure S1; Figure S2; Table S1
