# Supplementary material for: Comparative NanoUPLC-MSE analysis between magainin I-susceptible and -resistant Escherichia coli strains
Source: Sci Rep. 2017 Jun 23;7:4197. doi: 10.1038/s41598-017-04181-y (PMC5482854; doi:10.1038/s41598-017-04181-y)
Supplement: Supplementary file 1 — Figure S1; Figure S2; Table S1 [file 41598_2017_4181_MOESM1_ESM.pdf]

Comparative NanoUPLC-MS^E analysis between magainin I-susceptible and - resistant *Escherichia coli* strains

Marlon H. Cardoso[1, 2, 4 †], Keyla C. de Almeida[1, 2 †], Elizabete de S. Cândido[1, 4], André M. Murad[3], Simoni C. Dias[1] and Octávio L. Franco[1, 2, 4*]

[1]*Centro de Análises Proteômicas e Bioquímicas, Pós-Graduação em Ciências Genômicas e Biotecnologia, Universidade Católica de Brasília, Brasília- DF, 70.790-160, Brazil;*

[2]*Programa de Pós-Graduação em Patologia Molecular, Faculdade de Medicina, Universidade de Brasília, Brasília-DF, 70.910-900, Brazil;*

[3]*Embrapa Recursos Genéticos e Biotecnologia, Laboratório de biologia sintética, Parque Estação Biológica, Brasília-DF, 70.770-917, Brazil;*

[4]*S-Inova Biotech, Pós-graduação em Biotecnologia, Universidade Católica Dom Bosco, Campo Grande-MS, 79.117-900, Brazil.*

[†] These authors equally contributed to this article.

***Corresponding author:** Octávio Luiz Franco: Phone number: +55 61 34487167/ +55 61 34487220, Fax number: + 55 61 33474797, ocfranco@gmail.com

Supplementary Materials

Figure S1 - Uniformity of peptides between the two experimental conditions. The log_relative profile is the log of the fmol quantity of each peptide for each experimental condition (C1-3 and R1-3).

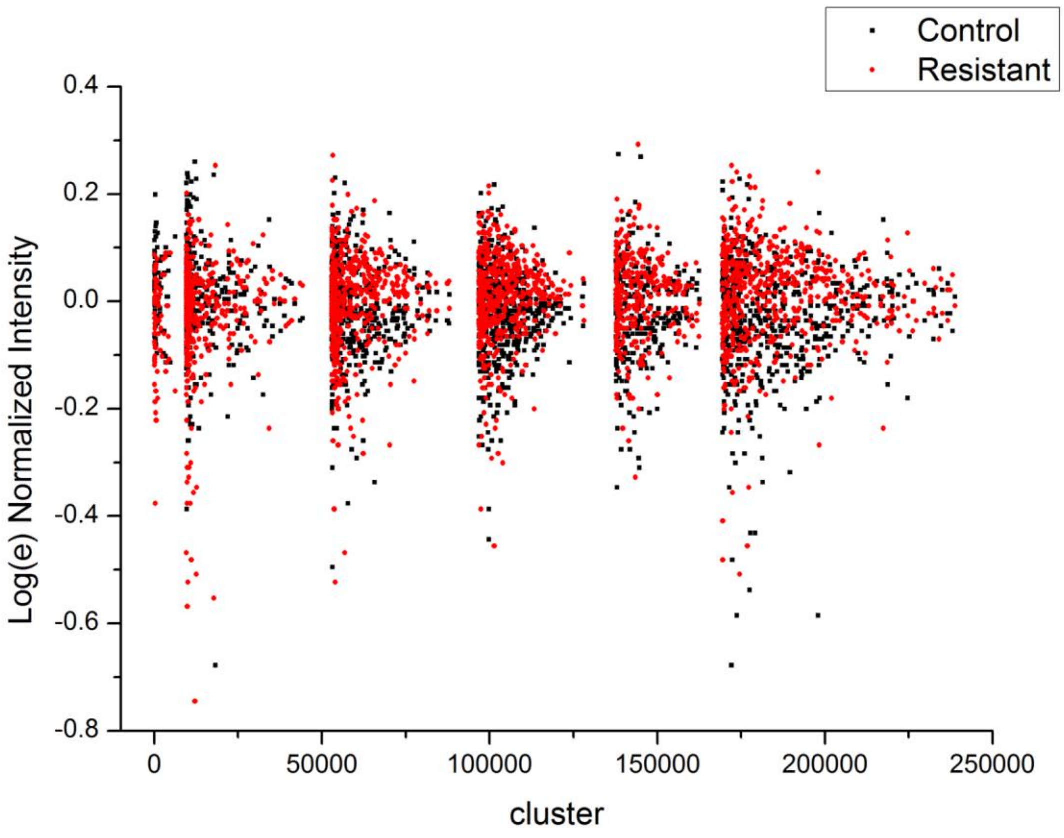

Figure S2 - *E. coli* downregulated and upregulated proteins. Magainin I-resistant *E. coli* differentially expressed proteins in comparison to –susceptible strains.

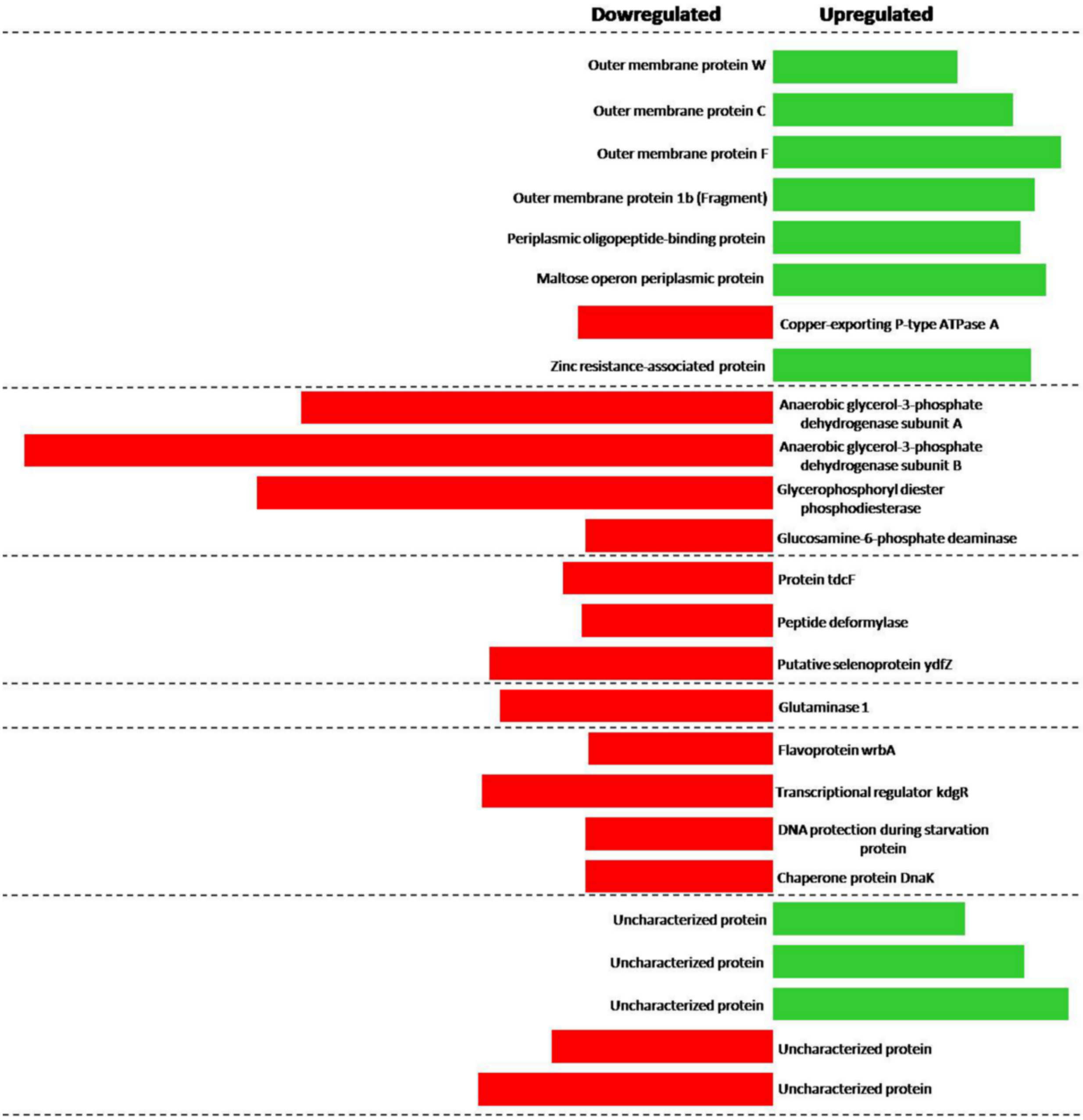

Table S1 – Exclusive proteins identified in the magainin I-resistant *E. coli* strains by nanoUPLC-MS^E.

UniProt identification	Score	Protein description	Biological processes
PIFC_ECOLI	353.62	Transcriptional repressor PifC	Transcription
DGOR_ECOLI	302.04	Galactonate operon transcriptional repressor	Transcription
RHAS_ECOLI	340.29	HTH-type transcriptional activator RhaS	Transcription
FRLR_ECOLI	423.25	HTH-type transcriptional regulator FrlR	Transcription
MODE_ECOLI	551.35	Transcriptional regulator ModE	Transcription
TYRR_ECOLI	201.23	Transcriptional regulatory protein TyrR	Transcription
NSRR_ECOLI	454.9	HTH-type transcriptional repressor NsrR	Transcription
METR_ECOLI	298.54	HTH-type transcriptional regulator MetR	Transcription
TTCA_ECOLI	345.25	tRNA 2-thiocytidine biosynthesis protein TtcA	Translation
TMCA_ECOLI	260.1	tRNA(Met) cytidine acetyltransferase TmcA	Translation

RSMI_ECOLI	254.85	Ribosomal RNA small subunit methyltransferase I	Translation
XNI_ECOLI	417.1	Flap endonuclease Xni	Replication
USPC_ECOLI	424.81	Universal stress protein C	Replication and repair
A5PFJ9_ECOLI	286.78	IS10 transposase	Replication and repair
RECJ_ECOLI	241.5	Single-stranded-DNA-specific exonuclease RecJ	Replication and repair
EXOX_ECOLI	448.56	Exodeoxyribonuclease 10	Replication and repair
EUTK_ECOLI	279.49	Ethanolamine utilization protein EutK	Transport and catabolism
FEPC_ECOLI	183.26	Ferric enterobactin transport ATP-binding protein FepC	Membrane transport
SECY_ECOLI	189.49	Protein translocase subunit SecY	Membrane transport
FHUA_ECOLI	244.6	Ferrichrome-iron receptor	Membrane transport
YADI_ECOLI	475.17	Putative phosphotransferase enzyme IIA component YadI	Membrane transport
ACRA_ECOLI	273.93	Multidrug efflux pump subunit AcrA	Membrane transport
NIKA_ECOLI	244.58	Nickel-binding periplasmic protein	Membrane transport

DPPA_ECOLI	273.55	Periplasmic dipeptide transport protein	Cellular motility
FLIG_ECOLI	283.19	Flagellar motor switch protein FliG	Cellular motility
YHGF_ECOLI	237.56	Protein YhgF	Cellular metabolism
YAEF_ECOLI	303.74	Probable endopeptidase YaeF	Cellular metabolism
CPTA_ECOLI	254.32	Toxin CptA	Cellular metabolism
FRSA_ECOLI	203.33	Esterase FrsA	Energy metabolism
MTLD_ECOLI	229.26	Mannitol-1-phosphate 5-dehydrogenase	Carbohydrate metabolism
GARD_ECOLI	174.22	Galactarate dehydratase (L-threo-forming)	Carbohydrate metabolism
MASY_ECOLI	240.63	Malate synthase A	Carbohydrate metabolism
MALQ_ECOLI	209.32	4-alpha-glucanotransferase	Carbohydrate metabolism
KDGK_ECOLI	255.59	2-dehydro-3-deoxygluconokinase	Carbohydrate metabolism
WCAE_ECOLI	388.26	Putative colanic acid biosynthesis glycosyl transferase WcaE	Metabolism and glycan biosynthesis
PPIC_ECOLI	720.19	Peptidyl-prolyl cis-trans isomerase C	Protein metabolism

HSLO_ECOLI	246.89	33 kDa chaperonin	Protein metabolism
TDCG_ECOLI	251.32	L-serine dehydratase TdcG	Amino acid metabolism
AK3_ECOLI	200.63	Lysine-sensitive aspartokinase 3	Amino acid metabolism
DDLB_ECOLI	280.3	D-alanine--D-alanine ligase B	Metabolism of other amino acids
PLSB_ECOLI	218.31	Glycerol-3-phosphate acyltransferase	Lipid metabolism
THIL_ECOLI	255.05	Thiamine-monophosphate kinase	Metabolism of cofactors and vitamins
PPNK_ECOLI	238.3	NAD kinase	Metabolism of cofactors and vitamins
RIBD_ECOLI	255.09	Riboflavin biosynthesis protein RibD	Metabolism of cofactors and vitamins
GUAC_ECOLI	235.03	GMP reductase	Nucleotide metabolism
NRDD_ECOLI	209.98	Anaerobic ribonucleoside-triphosphate reductase	Nucleotide metabolism
ENTB_ECOLI	353.76	Enterobactin synthase component B	Metabolism of terpenoids and polyketides
YNEI_ECOLI	217.2	Succinate semialdehyde dehydrogenase [NAD(P)+] Sad	Oxidation/Reduction

DHSB_ECOLI	228.58	Succinate dehydrogenase iron-sulfur subunit	Oxidation/Reduction
YKGC_ECOLI	199.51	Probable pyridine nucleotide-disulfide oxidoreductase RclA	Oxidation/Reduction
FDOG_ECOLI	220.31	Formate dehydrogenase-O major subunit	Signal transduction
STFR_ECOLI	195.66	Side tail fiber protein homolog from lambdoid prophage Rac	Host interaction
YOAB_ECOLI	376.62	RutC family protein YoaB	Biofilm formation
YJAH_ECOLI	416.62	Uncharacterized protein YjaH	Uncharacterized protein
YMFN_ECOLI	270.86	Uncharacterized protein YmfN	Uncharacterized protein
YAIV_ECOLI	269.7	Uncharacterized protein YaiV	Uncharacterized protein
YHIN_ECOLI	258.38	Uncharacterized protein YhiN	Uncharacterized protein
YHCC_ECOLI	420.17	Protein YhcC	Uncharacterized protein
YACC_ECOLI	467.77	Uncharacterized protein YacC	Uncharacterized protein
YCCE_ECOLI	351.62	Uncharacterized protein YccE	Uncharacterized protein
